# Estrogen in the male: a historical perspective^†^

**DOI:** 10.1093/biolre/ioy043

**Published:** 2018-02-09

**Authors:** Rex A Hess, Paul S Cooke

**Affiliations:** 1 Department of Comparative Biosciences, University of Illinois at Urbana-Champaign, Urbana, Illinois, USA; 2 Department of Physiological Sciences, University of Florida, Gainesville, Florida, USA

**Keywords:** male reproduction, estrogen, testis, rete testis, efferent ductules, prostate

## Abstract

Estrogens have traditionally been considered female hormones. Nevertheless, the presence of estrogen in males has been known for over 90 years. Initial studies suggested that estrogen was deleterious to male reproduction because exogenous treatments induced developmental abnormalities. However, demonstrations of estrogen synthesis in the testis and high concentrations of 17β-estradiol in rete testis fluid suggested that the female hormone might have a function in normal male reproduction. Identification of estrogen receptors and development of biological radioisotope methods to assess estradiol binding revealed that the male reproductive tract expresses estrogen receptor extensively from the neonatal period to adulthood. This indicated a role for estrogens in normal development, especially in efferent ductules, whose epithelium is the first in the male reproductive tract to express estrogen receptor during development and a site of exceedingly high expression. In the 1990s, a paradigm shift occurred in our understanding of estrogen function in the male, ushered in by knockout mouse models where estrogen production or expression of its receptors was not present. These knockout animals revealed that estrogen's main receptor (estrogen receptor 1 [ESR1]) is essential for male fertility and development of efferent ductules, epididymis, and prostate, and that loss of only the membrane fraction of ESR1 was sufficient to induce extensive male reproductive abnormalities and infertility. This review provides perspectives on the major discoveries and developments that led to our current knowledge of estrogen's importance in the male reproductive tract and shaped our evolving concept of estrogen's physiological role in the male.

## Teaser

Estrogenic activity, which was first thought to be harmful to males, has now been shown to be produced locally in significant quantities and to be essential for male reproductive tract development and fertility.

## Introduction

It has been almost 90 years since estrogens were first isolated and identified historically as being associated with female reproduction. However, from the very beginning of this linkage to the female, there has been evidence that estrogens were also being produced in males [1]. Nevertheless, our understanding of the overall role of estrogens in male reproductive and nonreproductive organs has clearly lagged that in the female. In the last two decades, it has become clear that endogenous estrogen signaling is essential for male reproduction, due in large part to the availability of a number of knockout and transgenic animal models in which aspects of estrogen production or signaling had been altered. In this review, we discuss some key historical developments over the past century, with an emphasis on more recent research obtained from the various gene-targeted models that disrupt estrogen signaling or production. Our intent is to give perspectives on the major discoveries and developments that led to our current knowledge that estrogen and estrogen receptors (ER) regulate male fertility, efferent duct and prostate development and function, and in adults the flow of sperm from testis to the epididymis. Other reviews have already provided details on specific discoveries in the male [2–18], and the interested reader is referred to these for more in-depth discussions of specific aspects of this field.

## Early studies of estrogen and male reproduction

Our understanding of the role of estrogen in the male is inextricably linked to and dependent on our more general understanding of how estrogen works. The female hormone was first predicted in the 1920s by Edgar Allen and Edward Doisy. The first estrogen isolated and identified was estrone (E1), which was discovered independently by Doisy in St. Louis and by Butenandt in Germany [19, 20]. Doisy continued this work, and also discovered “dihydrotheelin” in 1940, which was later renamed 17β-estradiol (E2) [21]. These discoveries represent cornerstone events in this field. The terms “male and female hormones” were used in the literature throughout the 1920–30s, even though actual chemical compounds responsible for these actions had not yet been isolated [22]. Most of the initial studies on estrogen in males were focused on pathology caused by exposure of males to the “female hormone,” because it was unclear that this hormone was having a normal physiological function in the male. For example, Harold Burrows showed that treatment of adult male mice with estrogenic substances induced metaplasia in the coagulating glands and hyperplasia-like growth of epithelium and connective tissue in the prostate [22]. However, beginning in the 1940s, Charles Huggins described the beneficial effects of estrogens on prostatic cancer in men [23], work for which he received the Nobel Prize in Physiology or Medicine in 1966. Although it was not clear at the time, therapeutic estrogen treatments for prostatic cancer were later shown to induce their beneficial effects primarily by suppressing endogenous androgens that drive prostatic cancer growth. Nevertheless, these results made it clear that although estrogen was considered the female sex hormone and most often thought to be harmful in males, it clearly could have positive effects in certain clinical cases.

The prevailing early hypothesis was that “excess estrogen would produce abnormalities in the male” [24]. It is interesting that as early as 1936, feminizing testicular tumors in the dog were found to produce estrogenic activity when transplanted into control males and females [25]. The source of this estrogen was later identified as coming from Sertoli cell tumors, which became a major focus at the time, because they were also associated with cryptorchidism and prostate abnormalities [26, 27]. Abnormalities induced by exogenous estrogens were even more pronounced when fetal or neonatal animals were exposed developmentally, with the formation of massive epididymal cysts and other male reproductive anomalies resulting from early estrogenization [28]. The work of McLachlan and collaborators [29, 30] demonstrated that exposure of developing rodents to high levels of estrogens, including the synthetic estrogen diethylstilbestrol (DES) or various natural or man-made environmental estrogens, could produce permanent changes in the structure and function of adult male reproductive organs. The DES studies in rodent models have been especially important because they predicted some of the extensive male abnormalities that have been reported in men exposed to DES during pregnancy [31–33]. However, there are significant differences in human and rodent responses to the synthetic estrogen, as DES does not alter testosterone production in fetal human testis as it does in the rat [34]. Probably, one of the more important long-term benefits from these early studies of DES was the recent conclusion that androgen receptor (AR) and estrogen receptor 1 (ESR1) expression in males must be carefully examined together and that these receptors and their respective steroid ligands should not be studied independently, as it is the balance between the two hormonal pathways that is important in the male, particularly during development [35]. However, much remains to be investigated, as others have now shown that DES effects on prostate development are clearly ESR1-dependent [36–38].

In addition to the discovery that Sertoli cell tumors produce feminization, extensive basic research on estrogen in the male was also being performed during the first half of the 20th century. References to the production of an estrogenic substance by the testis began as early as the 1920s [1], and for many years the measurement of estrogen in peripheral blood and urine of the male was of particular interest [39], and was frequently associated with efforts to determine the metabolites of testosterone [40–44]. The concentration of estrogen in peripheral blood was typically very low in males [45–48], but in horses estrone sulfate was found to reach as high as 2447 pg/ml [49]. Sizable quantities of estrogen were also found in testis [45, 49, 50] and in semen [51–53]. In semen of bulls, boars, and horses, conjugated estrogens were reported to range from 400 to 9000 pg/ml [54–57].

In all species examined, E2 is relatively high in testicular venous blood and lymph, but the highest concentration in the rodent male reproductive tract was found in rete testis fluid, which was reported to be 249 pg/ml [58]. Therefore, the source of testicular estrogen became the main focus for several laboratories. The primary source in immature testes was considered the Sertoli cell [59], a conclusion first drawn from work with Sertoli cell tumors, but later because in vitro studies for the first time showed direct evidence that normal Sertoli cells synthesize E2 under the regulation of follicle stimulating hormone and cyclic AMP [60]. However, in adult testes the consensus estrogen source was Leydig cells, which were thought to be the only testicular cell capable of estrogen synthesis [59, 61–67].

## Estrogen synthesis in testis

**Figure 1.**
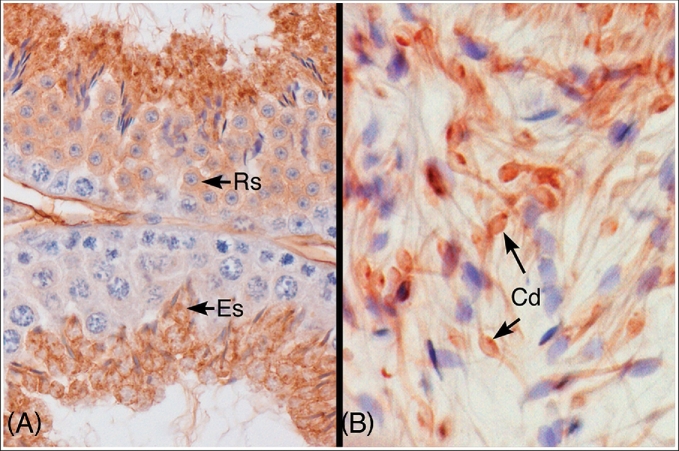
Immunohistochemical localization of P450 aromatase protein in the mouse testis and epididymis. (A) Aromatase protein was expressed in the cytoplasm of round (RS) and elongated spermatids (ES) in the mouse seminiferous epithelium. (B) Caput epididymal lumen. Aromatase protein was localized in the cytoplasmic droplet (Cd) and along the thin tails of the spermatozoa. Used with Open Access permission from a prior publication [11].

The paradigm that Leydig cells were the sole source of estrogen in adult testes remained intact for over 30 years [reviewed in 5, 6]. In hindsight, this seems logical, since it was well known that human chorionic gonadotropin stimulates Leydig cells in the male and increases peripheral and urinary estrogen concentrations [60, 61, 68, 69]. However, in 1992 a serendipitous discovery was made at the University of Illinois that revealed an additional source of estrogen in the testis and male reproductive tract [70–73]. These reports were the first to show that germ cells and epididymal sperm expressed P450 aromatase (CYP19A1) and actively synthesize estrogens from androgens (Figure 1). Aromatase was first localized by immunohistochemistry using an antibody that was among the best in the world at the time [74]. It was surprising to observe elongating spermatids with intensely positive immunoreactivity, because the literature clearly stated that only Leydig cells were positive in adult testes [60, 61]. To rule out a nonspecific reaction, staining was repeated and numerous controls were employed until the evidence was compelling, even though it contradicted decades of research. Finally, after obtaining western blot and mRNA data, as well as direct measurement of in vitro aromatase activity in isolated sperm, this iconoclastic discovery was published [70].

Looking back, it is easy to see why the presence of aromatase in spermatids was overlooked. Earlier work on testicular aromatase was performed before purification of the protein, which hindered the production of a specific antibody for immunohistochemistry, and cloning of the cytochrome P450 gene [13, 74]. Another major contribution to this discovery was a more widespread adoption of the STAPUT apparatus [75] for the separation of germ cells, which was later popularized by Marvin Meistrich and Irving Fritz [76, 77]. Although our data and that of others [6] have demonstrated greater aromatase activity in germ cells than interstitial cells (the archetype cell previously accepted as the sole estrogen source in adult testis), many papers today still downplay this discovery and refer to Leydig cells as the primary estrogen source in the male. Unfortunately, aromatase antibodies do not give equal specificity across species and also depend on good tissue fixation. Therefore, the literature continues to be inconsistent and contributes to the confusion.

## Localization of estrogen target cells in developing male reproductive tracts

Discovery of the receptor molecules responsible for estrogen-induced biological actions and establishment of their structure and locations were also critical to the nascent understanding of estrogen function in the male, as well as the female. The increased availability of radioisotopes after the Second World War provided biologists with new and powerful tools for tagging biological molecules. By following the distribution and metabolism of labeled molecules, it was possible to gain novel insights into important biological processes that had previously been intractable. As has been true throughout the history of biology, important methodological breakthroughs quickly translate into seminal discoveries in a myriad of biological disciplines. For example, beginning in the late 1940s, Melvin Calvin and coworkers used radioisotopes such as ^14^C to establish the basic pathway of photosynthesis [78].

The increased availability of radioisotopes for producing labeled biological molecules played a critical role in steroid endocrinology as well. Elwood Jensen and colleagues at the University of Chicago developed a method for labeling E2 with the radioisotope tritium (^3^H). They then injected this ^3^H-E2 into rats, and followed its localization and metabolism [reviewed in 79, 80]. Their data on the distribution and metabolic fate of labeled E2 led to the establishment of our modern concept of ER. An important observation from these studies was that the estrogen molecule injected in vivo remained intact. Equally as important was the finding that the radiolabeled ^3^H-E2 did not distribute homogeneously within the injected animals, but instead became concentrated in certain organs such as the uterus. Trophic effects of the ovary and estrogen on various aspects of uterine structure and function had been known for years, and it became obvious that localization of labeled ^3^H-E2 correlated with its biological effects. Subsequent work involving purification of estrogen-bound molecules in the uterus by Gorski, Jensen and others then led to the identification of the original ER (ESR1, also called ERα), the eventual determination of its structure and mechanism of action [81, 82] and finally the cloning of the molecule by Chambon and colleagues [83, 84].

Although descriptions of estrogen effects in males go back to the 1930s [85], it was not until decades later that the pioneering work of Elwood Jensen showing preferential binding of ^3^H-E2 in certain organs [82] laid the groundwork for our understanding of ER localization in male reproductive tissues. The initial methodology of Jensen that identified preferential distribution of labeled estrogen provided no information regarding specific cell types or regions in an organ that concentrated the radiolabel and thus contained the presumptive ER. However, studies using isolated areas of organs (e.g., hypothalamus of the brain) or specific regions (epididymis) or cell types (e.g., interstitial cells in the testis) made it clear that ER were widely distributed in the male, including in brain, reproductive tract and other organs such as the liver [86, 87]. Studies in the 1970s and 80s unmistakably identified ER protein in the testis, male reproductive tract and accessory sex organs, using ^3^H-E2-binding methods [66, 88–93]. Early work with radiolabeled E2 also led to the advent of steroid autoradiography, which was developed in part by Walter Stumpf and Madhabananda Sar at the University of North Carolina-Chapel Hill [94, 95]. This technique allowed mapping of the histological distribution of ER in an organ, and studies from these investigators were among the first to reveal high expressions of ER in developing male reproductive tissues, which suggested a mechanistic basis for the already well-known deleterious effects of early estrogen administration. The method was also useful in identifying estrogen targets in organs that expressed relatively low amounts of ER or that expressed ER in a limited number of cell types, especially during organ development.

Steroids are labile in tissue, and thus it was not a simple task to use radiolabeled steroids to examine their distribution in histological sections. These problems were overcome using a technique in which tissues were exposed to radiolabeled estrogens, such as ^3^H-E2, either in vivo or in vitro, and then flash-frozen at the end of the incubation. The tissues were then sectioned, again in the cold to retain the labeled steroid in place, and dry-mounted onto glass slides previously coated with photographic emulsion [94]. After a variable incubation period, which was dependent on the ER concentration in a target tissue and the specific activity of the radiolabel, the slides were developed and stained. By examining where steroids such as ^3^H-E2 had localized, the pattern of ER expression in that tissue could then be determined at the cellular level. This technique was widely used in organs such as brain and various adult male and female reproductive organs [96].

Because this method allowed ER to be identified in even the tiniest organs, it was used to establish the distribution of E2 in fetal and neonatal male tissues. Extensive expression of ER in reproductive and nonreproductive tissues of fetal and neonatal mice was reported [97]. Expression of ER in the Mullerian and Wolffian ducts and the urogenital sinus of the developing male reproductive tract was abundant, and was also seen in late fetal and neonatal reproductive organs derived from these structures [97].

The extensive data showing ER expression in developing male organs helped to change the focus from harmful effects of estrogen in the male to a new hypothesis suggesting that testicular estrogen might promote normal development and function in the reproductive tract, especially in efferent ductules. The discovery of aromatase in testicular germ cells and sperm facilitated this change in focus. A paper from the laboratory of Sar [98] reported that in the adult, efferent duct epithelial cells were labeled more heavily with ^3^H-E2 than with ^3^H-DHT. Other regions of the epididymis were also positive for E2, but the efferent ductules were “remarkably higher.” Therefore, a collaborative effort was made by Cooke and Hess to examine whether this unique labeling of efferent ducts was also present during development. Indeed, it was found that ER binding, along with AR, was detected in efferent ductule epithelium by day 16 of gestation [99]. These small but distinctive ductules were the first epithelial tissue in male reproductive organs to express ER and also had the highest level of E2 binding in the developing male. This study was the first to identify *developing* efferent ductules as a major estrogen target and they uniquely expressed high ER levels beginning early in development and continued through adulthood. High levels of ER expression in this tissue suggested that estrogens might have a functional role there, a harbinger of subsequent studies that identified the first physiological role for ESR1 in the male reproductive tract [100].

**Figure 2.**
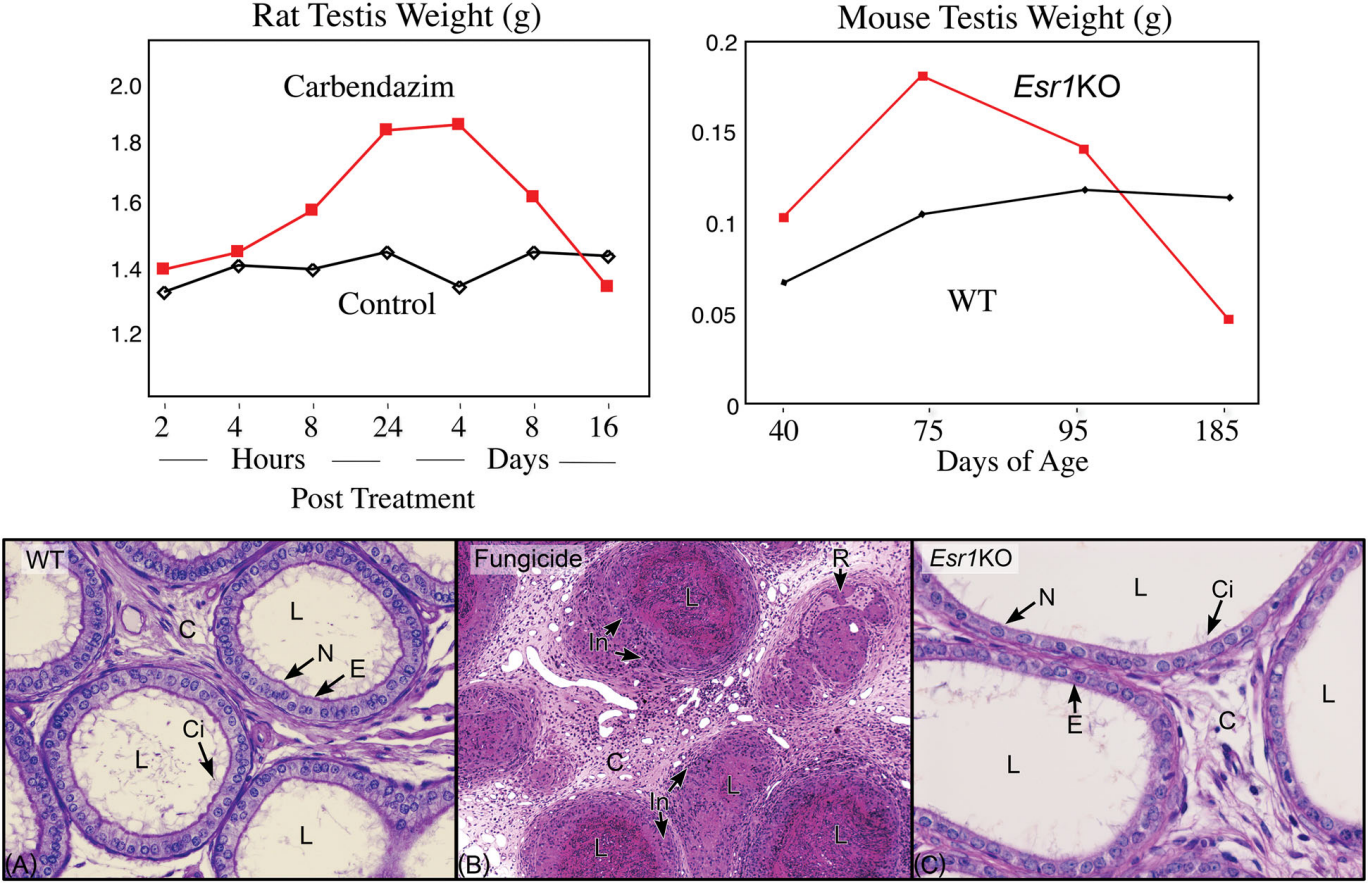
Comparison of the effects on testis weight and efferent ductules after treatment with the fungicide carbendazim and in the *Esr1* knockout mouse (*Esr1*KO). Modified with permission from a prior publication [100]. On the left, rat testis weight increases within hours after treatment with carbendazim (red), reaching a peak by day 4 and then decreasing as the testis atrophies. On the right, testis weight increases in *Esr1*KO mice (red) reaching a peak on day 75, but then decreasing until it has atrophied by day 185. In both cases, testicular atrophy was preceded by increased testis weight due to back-pressure of fluid accumulation caused by disruption of efferent ductule structure and function. (A) Wild-type (WT) efferent ductules showing a normal lumen (L) containing mostly fluid that must be reabsorbed as sperm are transported toward the epididymis. The epithelium (E) has a normal height and is lined by ciliated cells (Ci) with long motile cilia extending into the lumen and by nonciliated cells (N) that have a periodic acid-Schiff's positive border at the lumen where numerous microvilli are present. The connective tissue (C) contains loosely scattered fibroblasts and blood vessels. (B) Efferent ductules from the fungicide carbendazim-treated rat. The lumens (L) are occluded with coagulated sperm and cellular debris. The lining epithelium cannot be distinguished at this magnification due to the number of inflammatory cells (In) surrounding the ductules in the densely populated connective tissue (C), which also appears thickened. In one area, the lining epithelium appears to have begun recanalization (R) or regrowth around the occlusion. (C) *Esr1*KO efferent ductules showing a wider lumen (L) due to dilation and fluid accumulation. The epithelium (E) is shorter in height, but lined by ciliated (Ci) and nonciliated (N) cells. The connective tissue (C) appears similar to that in the WT. The cilia appear to be shorter and fewer in number compared to WT. Apical cytoplasm in the nonciliated cells is scarce and the microvillus border is missing in many areas.

Efferent ductules are delicate, coiled tubules that connect the rete testis with the epididymis. In rodent species, these ducts are found within the epididymal fat pad, merging until they form a single tubule that enters under the epididymal capsule [11, 101]. In larger mammals, the ducts occupy up to nearly 50% of the caput and enter the epididymis as individual tubules, mostly without merging. Their function is to transport sperm rapidly, while increasing sperm concentration nearly 28-fold, using a kidney-like mechanism of fluid reabsorption [102]. Early on it was discovered that the efferent ductule's role in fluid reabsorption was very important and that disruption of this function could lead to occlusions, which often resulted in testicular fluid buildup [103, 104]. Various chemicals induce blockage of the ductules, including the benzimidazole fungicides, benomyl, and the metabolite carbendazim. A single dose of these fungicides causes efferent ductule blockage and prevents sperm transport in rats [12, 105]. As with ligation of the proximal region [104], occlusions result in testicular atrophy due to fluid accumulation, which backs up into the rete testis and causes a temporary increase in testis weight (Figure 2). Unbeknownst at the time, this discovery of the unique testicular response to efferent ductule pathology would become the basis for uncovering the first physiological function for estrogen in the male reproductive tract (Figure 2).

## ESR1 and ESR2 in the adult male reproductive tract

Early ^3^H-E2 binding studies provided strong evidence for ER specificity and expression in the male reproductive tract [66, 88–93]. However, until the mid-1990s, most scientists did not believe estrogen played a major role in the male, or at best functioned primarily during development, while in the adult, estrogen binding was suggested to be residual activity [106, 107]. Also during that time period, it was assumed that binding of labeled E2 to targets in all organs, male or female, was due to the presence of only one ER type. The discovery of a second ER, ESR2, by Jan-Åke Gustafsson's laboratory in 1996 immediately changed that assumption [108]. After that discovery, all previous work based on E2 binding to putative ER protein required re-examination, to determine which receptor was expressed and in which specific cells. As good antibodies became available, localization of both ESR1 and 2 became a priority, and immunohistochemical studies were supplemented with other methods, including in situ hybridization and northern blot analysis.

**Figure 3.**
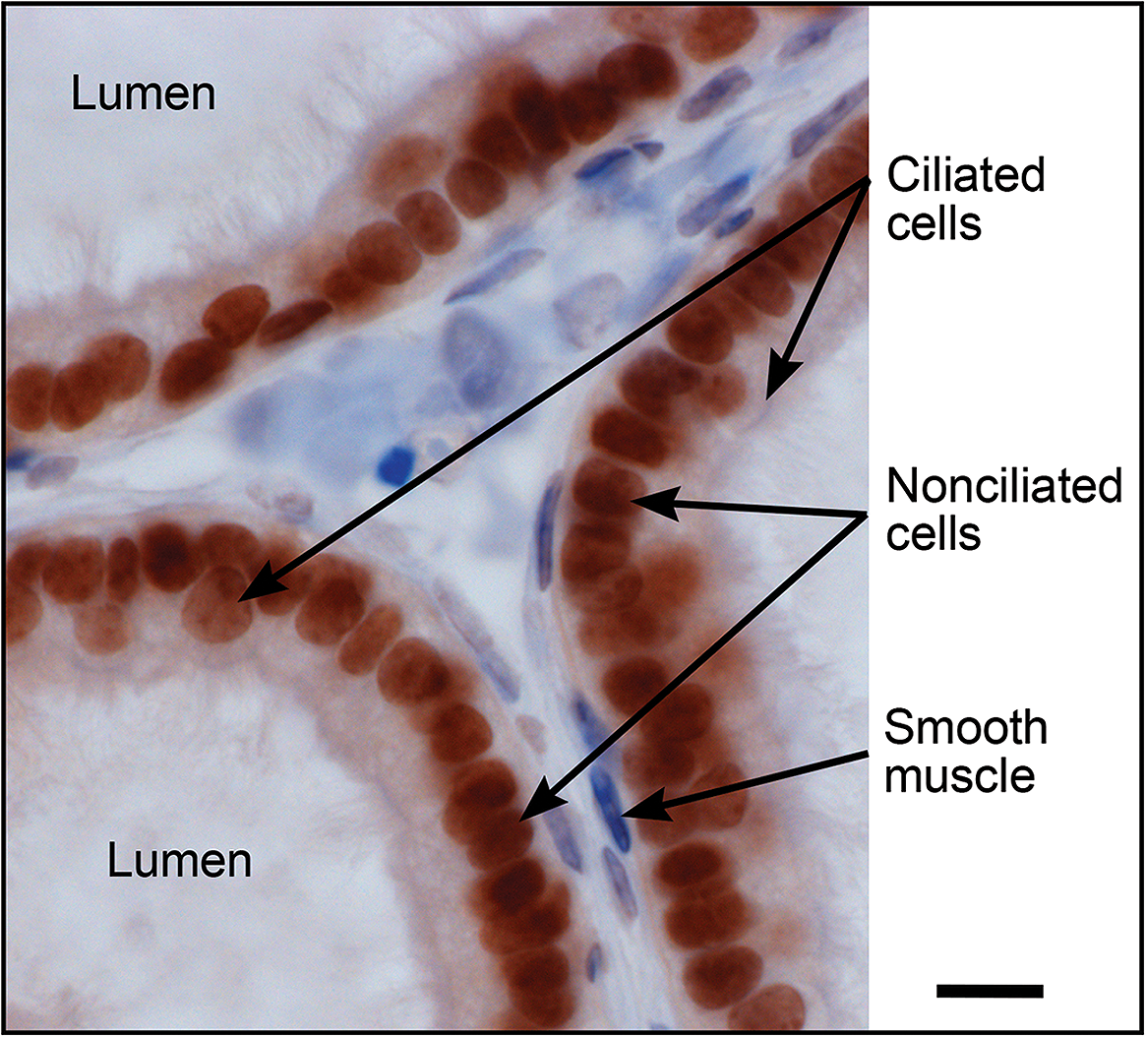
ESR1 immunostaining with 6F11 antibody (NCL-ER-6F11; Novocastra, Newcastle upon Tyne, UK) in mouse efferent ductules. Ciliated and nonciliated cells are both ESR1 positive, but nonciliated cell nuclei are more intensely stained. Most peritubular smooth muscle cells are negative. The cytoplasm of all epithelial cells shows a slightly positive reaction, which would be consistent with recent data revealing the importance of membrane ESR1 in maintenance of efferent ductule structure and function [140]. Bar = 10 μm.

Unfortunately, efforts to localize ESR1 and 2 have not provided consistent results in the male and discrepancies occur throughout the literature [4, 7, 14, 109], similar to research on aromatase in the male. These inconsistencies are most likely due to unequal antibodies, variations in biochemical methods, as well as species differences. Literature on ESR1 immunostaining in testis and epididymis has been particularly contradictory, with Sertoli and germ cells showing the greatest variations and the principal cells of the epididymis being negative in some studies, while showing distinct cytoplasmic and nuclear staining in others [7]. However, recent studies indicate that we may need to look more closely at estrogen function in testis and epididymis, as it appears to have a role in spermiogenesis [14, 110, 111], as well as maintenance of some aspects of epididymal physiology [7, 112–114]. Nevertheless, across species and laboratories ESR1 has been consistently abundant in efferent ductule epithelium, and ESR1 immunohistochemical staining is more intense there than in any other tissue, male or female (Figure 3). The expression of ESR1 mRNA is approximately 3.5-fold greater in efferent ducts than in the female uterus [115]. In efferent ductules, however, it remains to be determined why ciliated cells are ESR1-negative or show reduced expression in some species, especially in light of recent studies showing an *Esr1*KO-like phenotype when ciliated cell development is inhibited [116].

At first ESR2 protein and mRNA appeared to be expressed ubiquitously in the male reproductive tract [117–120]. However, more recent studies have raised doubts regarding antibody specificity for immunohistochemical localization of the receptor [14, 121]. For example, the S-40 and PA1-311 antibodies for ESR2 stained testicular germ cells, but in the *Esr2*RFP mouse (in which an iCre insertion into *Esr2* drives the RFP transgene) only interstitial cells were positive [14], which is consistent with results using the highly specific antibody PPZ0506 [121]. Additionally, the difficulty of studying ESR2 is amplified when comparing expression across organs. Results with the PPZ0506 antibody suggested that ESR2 was not expressed in prostate [121], while the *Esr2*RFP mouse prostate exhibits one of the strongest signals for *Esr2*-driven expression of RFP [14]. Therefore, previous data on ESR2 expression and specific localization need re-evaluation, especially in light of the failure by some to observe a male reproductive phenotype in *Esr2*KO mice, while others show changes in the developing testis and the aging prostate (Table 1).

**Table 1.** Male reproductive phenotypes in estrogen pathway gene targeted and transgenic animal models.

Models	Names	Descriptions	Key Phenotypes	References
*Esr1*(ERα)-null mouse	αERKO ERαKO Ex3αERKO	Global deletion of *Esr1*	Males infertile; disrupted mating behaviors; increased T; +/– increased LH Testis: normal at birth followed by transiently increased testis wt; dilation of seminiferous tubules followed by testis atrophy; loss of spermatogenesis with aging; sperm flagellar coiling in lumen; transplanted spermatogonia produce normal sperm Rete testis: overgrowth and dilation Efferentductules: luminal dilation and dysmorphogenesis; decreased epithelial height, fluid reabsorption, endocytic apparatus and ciliary number and size; decreased SLC9A3, *Slc9a3,* AQP1,9 and CAR2; increased CFTR; increased number of blind-ending tubules; glycogen granules in proximal cells Epididymis: abnormal narrow and clear cells; abnormal growth of epithelium in efferent duct regions; sperm granulomas; decreased SLC9A3, CAR14 and SLC4A4; decreased sperm motility; decreased luminal osmolality; increased luminal pH; increased acrosome-reacted sperm; sperm flagellar angulation and coiling Prostate: no effect	[2, 38, 100, 112, 113, 124, 125, 127, 128, 142, 171, 193, 206–212]
*Esr1*(ERα)-conditional deletion mouse	ACTB-ERαKO	Global deletion of *Esr1* using β-actin-Cre	Males infertile; increased T Testis: decreased wt Epididymis: decreased sperm number Prostate: decreased branching morphogenesis and fibroblast proliferation	[213]
*Esr1*(ERα)-conditional deletion mouse	fERαKO	Crossed floxed ERα with fibroblast-specific protein (FSP)-Cre	Males fertile Testis: normal Epididymis: normal Prostate: decreased wt; decreased branching morphogenesis; increased stromal apoptosis; no effect on *AR* and *Esr2*; increased *Bmp4*DES treatment: prostate hyperplasia	[198, 214, 215]
*Esr1*(ERα)-conditional deletion mouse	PesERαKO	Crossed floxed ERα with probasin (prostate epithelial)-Cre	Males fertile Testis: normal Epididymis: normal Prostate: normal DES treatment: inhibited prostate hyperplasia	[214]
*Esr1*(ERα)-conditional deletion mouse	SmERαKO	Crossed floxed ERα with Tgln (SM22α smooth muscle)-Cre	Males fertile Testis: normal Epididymis: normal Prostate: less folding; abnormal growth; decreased basement membrane thickness; no effect on branching	[198]
*Esr1*(ERα)-conditional deletion mouse	dERαKO (fERαKO + SmERαKO)	Crossed Tgln-cre with FSP-cre; then crossed offspring with floxed ERα to create double knockout	Males fertile Testis: normal Epididymis: normal Prostate: compound phenotype of both fERαKO and smERαKO	[198]
*Esr1*(ERα)-null rat	Ex3αERKO	Global deletion of *Esr1*	Males infertile Testis: at 10 weeks, decreased wt; seminiferous tubule dilation; Efferent ducts: not examined Epididymis: traces of sperm in cauda	[145]
*Esr1/2* (ERα/ERβ)-null	Ex3ERαβKO DERKO	Global deletion of *Esr1* + *Esr2*	Males infertile; seminiferous tubular luminal swelling; reduced cauda epididymal sperm density; reduced sperm motility	[193, 216]
*Esr2* (ERβ)-null	βERKO	Global deletion of *Esr2*	Males fertile Testis: normal Epididymis: normal and normal sperm motility Prostate: increased prostate lymphocytic infiltrate; epithelial hyperplasia with aging	[36, 37, 147, 148, 182, 193, 216–218]
Esr2(ERβ)-null	Ex3βERKO	Global deletion of *Esr2*	Increased gonocytes 2 dpp; increase gonocyte proliferation; decrease gonocyte apoptosis; normal number Sertoli and Leydig cells	[38]
*Esr2* (ERβ)-null	βERKO Ex3βERKO	Global deletion of *Esr2*	Males fertile; normal testis, epididymis and sperm motility	[148]
ESR1 overexpression	ESR 1+	Transgenic ESR1 overexpression under tetracycline control and doxycycline inhibition	Males fertile; normal T Testis: normal histology Seminalvesicle/coagulating glands: decreased wt Penis: no effect on AR; decreased skeletal muscle wt	[219, 220]
*Esr1* LBD mutant	ENERKI	Estrogen nonresponsive ERα knock-in; ERα agonist PPT activates mutant G524L ERα	Males subfertile; rescued by PPT; increased T and LH; testis degeneration with aging; normal testis to 12 weeks; sperm counts decreased after 20 weeks; normal efferent ducts	[176, 177]
DBD mutant	NERKI, αERKO (-/AA; AA), KIKO, ERα^AA/−^	DBD mutation on ERα-null background; precludes direct binding to ERE; thought to permit nonclassical ERα; however, DNA binding preference change from ERE to HRE	Males fertile; normal T, LH and FSH; normal testis weight; occasional dilated seminiferous tubule but mostly normal; abnormal testis with aging; partial decrease SLC9A3, normal sperm count; decreased Aqp1 delayed; normal Aqp9. Delayed, diminished or reversed αERKO male effects	[143, 221–224]
DBD mutant	EAAE	ERα DBD mutation (4 ERE sites); completely inhibited binding to ERE and HRE motifs	Males infertile; phenotype similar to αERKO; male reproductive tract not shown	[224, 225]
AF-1 mutant	ERα AF-1^0^	Deletion of AF-1 (ligand-independent)	Males infertile; male reproductive tract not shown	[226, 227]
AF-2 mutant	ERα AF-2^0^	Deletion of AF-2 (LBD)	Males infertile; male reproductive tract not shown	[228]
AF-2 mutant	AF2ER (KI/KI)	AF-2 Mutation; ICI182780 and tamoxifen (TAM) agonist through AF-1	Males infertile; testis seminiferous tubule dilation, slightly delayed from αERKO; increased T but not LH; dilated rete testis and efferent ductules; decreased SLC9A3 and AQP9; decreased *Slc9a3, Aqp9, Car2,* and *Aqp1*; effects reversed by TAM	[144]
*Esr1* D-domain mutant	H2NES	*Esr1* D-domain Hinge 2 mutation with nuclear export signal; cytoplasmic ESR1 only (nuclear ESR1 absent); possible post-translational problems	Males infertile; similar to αERKO; testicular atrophy; efferent ductules not shown	[229, 230]
Nuclear-only ESR1	NOER	Mutant lacking membrane localization of ESR1, but retains functional nuclear ESR1; palmitoylation site (cysteine 451) ESR1 mutant	Adult males infertile, but juvenile males subfertile; increased T Testis: increased wt at 4 mo; dilated seminiferous tubules and tubular atrophy; decreased DSP; coiled sperm tails in tubular lumens Rete testisand efferent ducts: dilated lumens Efferent ductules: abnormal epithelium similar to αERKO Epididymis: cauda sperm abnormalities; decreased sperm motility Seminal vesicle/coagulating glands: increased wt	[140, 195]
Membrane-only ESR1	MOER	*Esr1* LBD fused to transgene containing multiple palmitoylation sites on an *Esr1* knockout background	Males infertile; male reproductive tract not shown	[231]
Aromatase-null	ArKO +/– soy free	Global deletion; targeted exon IX of *Cyp19*	Males have decreased fertility due to impaired mounting behavior; aging effects on testis; normal efferent ducts; normal expression of *Esr1*, *Esr2* and *Slc9a3*	[142, 167, 169–171]
Aromatase over-expression	Int-5/aromatase; ARO M+	Transgenic male overexpression	Males subfertile to infertile; testis wt decreased (AROM+), 50% increased wt (Int-5/aromatase) Testis: Leydig cell hyperplasia/hypertrophy and tumors; abnormal spermatogenesis; decreased serum T, increased estradiol; increased ESR1; increased cyclin D1	[232, 233]
GPER1-null	GPERKO	Deletion of GPER1	Males fertile; no male reproductive phenotype; male reproductive tract not shown	[234, 235]
Estrogen sulfotransferase -null	ESTKO	Global deletion	Males fertile; testicular effects with aging; Leydig cell hypertrophic and hyperplastic; increased wt of seminal vesicles; decreased sperm motility with aging	[236]

^1^Abbreviations: wt, weight; mo, months; ESR1, estrogen receptor 1; ESR2, estrogen receptor 2; AR, androgen receptor; T, testosterone; FSH, follicle stimulating hormone; 4,4’,4'-(4-Propyl-[1H] pyrazole- 1,3,5-triyl), PPT; SLC9a3, sodium/hydrogen exchanger 3; AQP, aquaporin; CAR2 and 14, carbonic anhydrase 2 and 14; SLC4A4, sodium bicarbonate cotransporter; CFTR, cystic fibrosis transmembrane conductance regulator; GPER, G protein-coupled estrogen receptor 1; *Cyp19,* aromatase; LBD, ligand binding domain; DBD, DNA binding domain; HRE, hormone response element, ERE, estrogen response element; ARE, androgen response element; AF-1 and -2, activation functions 1 and 2 domains; DSP, daily sperm production; dpp, days postpartum

## Estrogen receptor signaling pathways and efferent ductule structure and function

Cloning of the human ER [122] and development of an ER antibody (the first for any steroid hormone receptor) [123], as well as the recognition that efferent ductules were a major target for estrogen in the male [95, 99] were historical landmarks leading up to the discovery of an essential role for ESR1 in the ductule physiology [100]. However, it was the generation of the first steroid receptor knockout model (Table 1), the *Esr1* knockout mouse (*Esr1*KO), that directly led to discovery of this unique function for the estrogen signaling pathway in males [124]. Although initial descriptions of *Esr1*KO males indicated that their testes were atrophic at maturity [125], our first experience with these males revealed that during early adulthood the testes showed a transient increase in weight, prior to regression of the seminiferous tubules [100]. Therefore, based on prior experience with fungicides that targeted efferent ductules and also produced a transient increase in weight (Figure 1), it was hypothesized that loss of ESR1 could be producing an occlusion of the ductules, causing fluid back up into the testis and dilating the rete testis lumen. However, contrary to this assumption, initial histological slides from *Esr1*KO males revealed no occlusion, but instead extreme ductal dilation, with large open lumens filled with excessive fluid and a greatly reduced epithelial height. Additionally, seminiferous tubules were dilated and testes showed a transient increase in weight followed by atrophy, a pattern previously observed with ductal occlusions after fungicide exposures [11].

**Figure 4.**
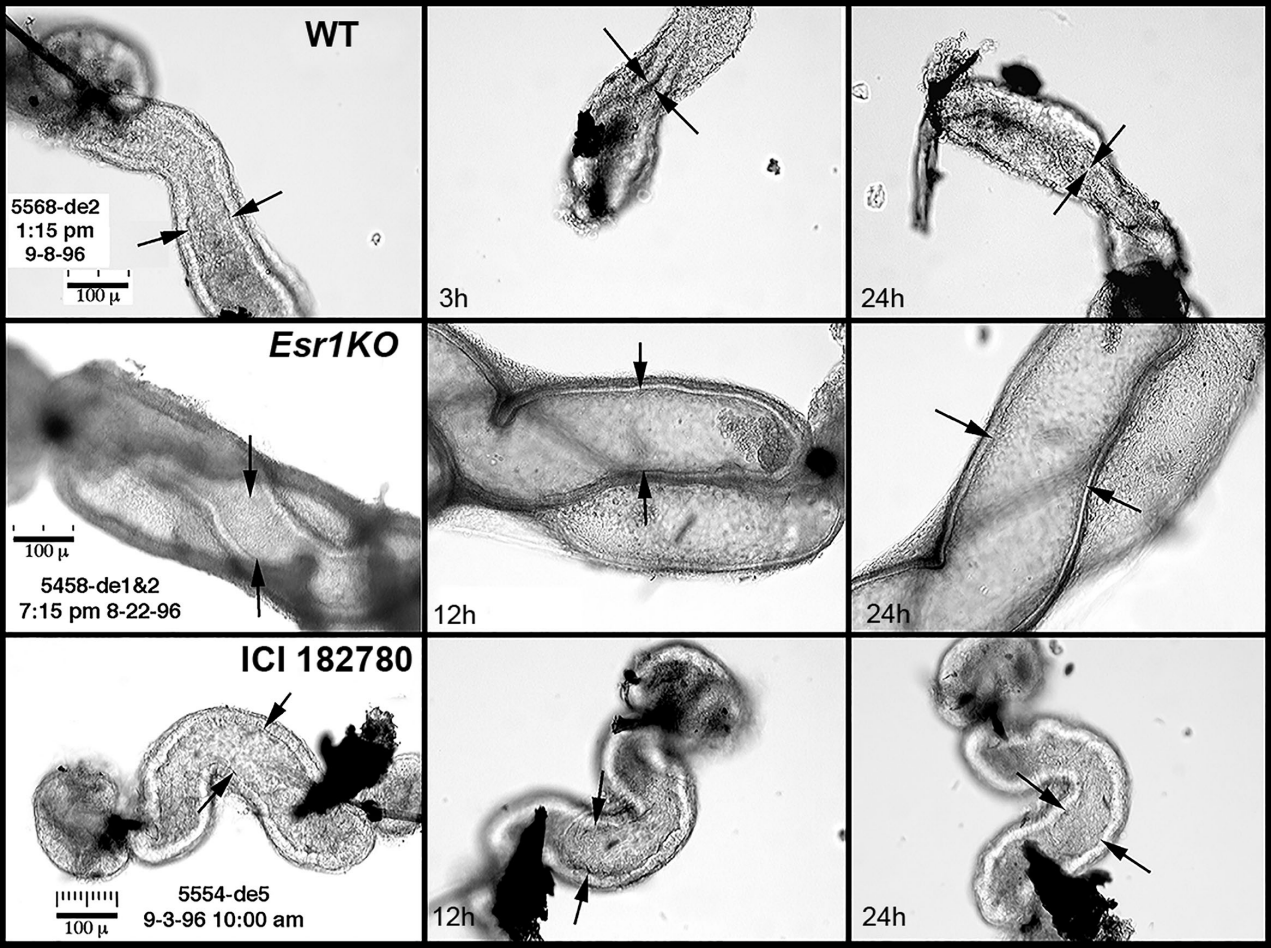
In vitro observations of efferent ductule segments after ligation (left to right). The lumens are indicated by the spaces between the arrows. The wild-type (WT) ductule lumen collapsed by 3 hours (h) and remained closed at 24 h, indicating that luminal fluids had been reabsorbed. The *Esr1*KO ductule lumen was wider than the WT and increased in diameter after incubation for 12–24 h, indicating that not only was fluid reabsorption inhibited, but fluid was being secreted into the lumen. The ductule from a male after receiving treatment for 3 days with the anti-estrogen ICI 182780 showed an essentially normal luminal diameter throughout the incubation period, indicating an inhibition of fluid reabsorption. Scale bars = 100 μm. These data are from an experiment previously published [100].

Some of the most difficult data to collect in the initial studies of *Esr1*KO efferent ductules, but possibly the most illuminating, involved in vitro incubations of small, ligated segments of isolated efferent ductules from wild-type and *Esr1*KO mice (Figure 4). These ductules are approximately 100–150 μm in diameter and very delicate, making them difficult to manipulate with fine forceps and nearly impossible to ligate with suture. Only after unraveling 0000 sutures in ethanol was it possible to tie a small loop to effectively ligate these structures. The thinner strands of looped sutures were then quickly moved to a buffer solution, and the ends of individual ductal segments pushed through the small loops and forceps were used to tighten and close the ends. It was assumed that control ducts would collapse over the next 12 h in vitro, as fluid was reabsorbed from the lumen, which indeed occurred. However, cultured *Esr1*KO ductules, which were already expanded, further dilated in vitro, indicating that fluid reabsorption was not only inhibited, but was also actively being transported into the lumen. Thus, our hypothesis quickly became focused on the concept that ESR1 regulates fluid reabsorption in efferent ductule epithelium.

**Figure 5.**
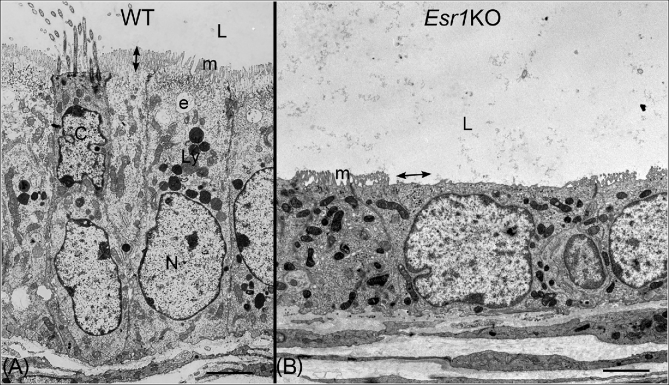
Transmission electron microscopy of efferent ductule epithelium in wild-type (WT) and *Esr1* knockout mice (*Esr1*KO). Bars = 7 μm. (A) WT efferent ductule showing ciliated (C) and nonciliated (N) cells lining the lumen (L). Nuclei of ciliated cells are found closer to the lumen. Cilia protrude into the lumen from a basal body located at the surface of the cell. Apical cytoplasm of nonciliated cells is filled with organelles associated with the endocytic apparatus (e), which sits just below the microvillus border (m), and has an abundance of lysosomes (Ly). Height of the microvilli is indicated by the double-headed arrow. (B) *Esr1*KO mouse efferent ductule showing greatly reduced epithelial height and loss of cilia, apical cytoplasm, and the endocytic apparatus. Microvilli (m) are shorter and missing in some areas (double-headed arrow). The mitochondria also have a much darker staining matrix than seen in the WT.

Although the knockout methodology has been a vital contribution to biomedical research, resulting organ phenotypes in global knockout mice always raise questions regarding a potential difference in gene requirements during development versus in the mature animal. In the *Esr1*KO, it was unclear from initial results whether the adult phenotype was simply a developmental defect or reflected the loss of genuine adult E2 actions. Therefore, the potent (pure) anti-estrogen [126] ICI182780 (ICI; Fulvestrant, sold under the trade name Faslodex) provided the ideal experimental tool for testing whether ESR1 and/or ESR2 are essential for fluid reabsorption in efferent ductules. Without the generous gift of this anti-estrogen from AstraZeneca (Macclesfield, UK), this research would have been delayed for years, as other less potent anti-estrogens give inconsistent and less convincing results. Efferent ductules from adult mice treated with ICI and incubated in vitro, after ligation of both ends, never collapsed (Figure 4), demonstrating that disruption of adult ESR1 signaling produced effects similar to that of the gene knockout from conception. This study provided a direct link between estrogen and the well-established physiological function of fluid reabsorption by the efferent ductule epithelium [100]. However, ICI-treatment studies and *Esr1*KO mice also confirmed another important conclusion. The epithelium of *Esr1*KO mice never exhibited differentiation. The epithelium is reduced in height at birth (unpublished observation) and remains undifferentiated throughout postnatal development and into adulthood, lacking mature microvilli and organelles in the apical cytoplasm (Figure 5), which are required for fluid reabsorption [127, 128].

Adult mice and rats treated with ICI have an undifferentiated efferent ductule epithelium, with reductions in epithelial height and number and size of microvilli, and loss of lysosomes [129–131]. However, a full anti-estrogen response requires between 50 and 100 days, although increases in luminal diameter begin within 8 days. Mice respond more rapidly than rats [129, 130], indicating species differences in estrogen sensitivity. Large genetic variation in E2 sensitivity was previously shown in mice and appears to be due to differences in estrogen metabolism through sulfotransferase activity [132]. Others have shown the expression of this steroid metabolizing enzyme in testis, epididymis, and prostate [133–139]. Thus, development of the epithelium requires ESR1 expression, but maintenance of the differentiated adult epithelium appears to involve more than just the activation of a nuclear ESR1 (nESR1), as loss of the membrane receptor (mESR1) also results in the *Esr1*KO phenotype [140]. The ability of ICI to inhibit fluid reabsorption, apparently through direct inhibition of ion transporters, causes a rapid build-up of luminal fluid, but morphological disruption requires a longer period of time and likely numerous other genes that are integrated with several hormonal and growth factor-activated pathways.

Since the first reports of *Esr1*KO mice were published [100, 124, 125], extensive research has been performed using these mice and several other animal models (Table 1). The major emphasis has been to uncover a molecular basis for estrogen's regulation of efferent ductule physiology. Because it was known that these ducts are derived from the embryonic kidney or mesonephros and resemble proximal convoluted tubules of the adult kidney [101], the first efforts examined molecular aspects of Na^+^ transport and H_2_O movement. The success of this research was due primarily to the rapid demonstration in *Esr1*KO males that Na^+^/H^+^ exchanger-3 (*Slc9a3*) was a key molecule that was downregulated in efferent ductules [141]. Further support of this idea came from *Slc9a3*KO mice, which also showed luminal dilations in both rete testis and efferent ducts, identical to *Esr1*KO males. However, epithelial morphology in *Slc9a3*KO ductules was normal, suggesting that key genes involved in maintenance of endocytosis, motile cilia, and the microvillus border were also regulated by ESR1 activity, as these were significantly reduced in the *Esr1*KO male, but not in the *Slc9a3*KO mice [100, 141]. ESR1’s regulation of this key gene has been consistent in all *Esr1*KO mice (Table 1) generated thus far [100, 112, 141–144], as well as in the ICI-treated adult mice and rats [129, 130].

Recently, *Esr1*KO male rats were generated and their reproductive phenotype resembled *Esr1*KO mice in that they were infertile with reductions in testis weight and cauda epididymal sperm [145]. However, *Esr1*KO rat males had lower testosterone concentrations, in contrast to *Esr1*KO mice, despite showing elevated LH levels. Also, closer examination of the published data reveal that the seminiferous tubules were not increased in diameter, as claimed in the paper, but rather the wider lumens may have resulted from the decreased height of the seminiferous epithelium. Thus, it is difficult to reconcile this tubular observation with the report that adult testes in *Esr1*KO rats were smaller in size. Maybe the difference in *Esr1*KO mice and rats is similar to the difference observed with anti-estrogen ICI treatments in these two species, as noted above [129, 130]. Additionally, an *Esr2*KO rat was recently reported [146]. In contrast to the females, which were infertile, the *Esr2*KO rat males were fertile and lacked reproductive tract abnormalities, which is consistent with most observations from *Esr2*KO mice [2, 147, 148]. However, we still need to reconcile the male *Esr2*KO data with those from other studies showing potential ESR2 functions (Table 1) in testis [38, 149–151] and prostate [14, 15, 152, 153].

Effects of adult anti-estrogen treatment on efferent duct epithelial morphology are complicated. It is unlikely that luminal dilation caused by downregulation of SLC9A3 (and thereby transcellular Na^+^ transport) causes epithelial flattening, as some suggested. Instead, ESR1 regulates numerous other structural genes. There is an over-representation of genes in efferent ductules that have estrogen response elements (EREs) in the promoter region [154, 155]. Gene overlapping peaks for EREs was 55% between efferent ductules and uterus, which means this male organ is enriched with genes also regulated by estrogens in females [155]. In contrast, the uterus overlaps with liver and aorta less than 20%. Thus, the importance of ESR1 in efferent ducts cannot be overemphasized. However, what is surprising, but also explanatory for the observed complexity, is recent data showing an abundance of expressed genes having both EREs and androgen response elements (AREs). *Slc9a3* (NHE3) and *Slc9a3R1* (NHERF1), the Na^+^/H^+^ exchanger and its major regulatory protein, which are located along the microvillus border of nonciliated cells, both have EREs and AREs in their promoters [154–156].

Sodium/hydrogen exchanger-3 is essential for water reabsorption in both efferent duct and kidney epithelium [157, 158] and both ESR1 and AR show high levels of nuclear colocalization in nonciliated cells of efferent ductules [118]. Thus, both receptors are capable of coregulation of numerous genes in this epithelium through direct ERE and ARE binding, respectively, as well as by tethering with common nuclear transcription cofactors [154, 155]. In light of these studies, epithelial cells may need the simultaneous presence of AR and ESR1 for adequate expression of genes such as *Slc9a3* that are essential to efferent duct fluid reabsorption. Such a requirement, in order to maintain physiological transport of Na^+^ across the epithelium, would help to explain prior studies showing that testosterone treatment of males increases the rate of fluid reabsorption, while E2 decreases reabsorption [159], which appears to contradict the initial findings in *Esr1KO* mice [115, 141]. However, subsequent studies have shown that exogenous E2 treatments downregulated both ESR1 and AR expressions, while androgen treatments had no effect on these steroid receptors in efferent duct epithelium [160]. Thus, without knowing the effects of steroid hormone dosing on the expression of relevant steroid receptors, the interpretation of physiological effects in the male reproductive tract could be misinterpreted.

Other studies also support the hypothesis that efferent duct epithelium requires both AR and ESR1 for balanced regulation of its physiology. For example, downregulation of ESR1, without effects on AR, results in efferent duct and rete testis luminal dilation in not only the *Esr1*KO mouse, but also in *Lgr4*KO [161, 162] and *E2f4/5*KO mice [116] and ICI-treated adult rodents [163]. Likewise, downregulation of AR, without effects on ESR1, also results in the same luminal dilations [164–166]. Thus, Richard Sharpe's conclusion that “…it is the androgen:estrogen balance that is of central importance in male reproductive development, as opposed to just androgens alone” is most apropos [35]. Efferent ductules are unique in requiring both AR and ESR1 expression for normal structure and the lack of either receptor alone causes luminal dilation.

Our original finding of aromatase expression by sperm promoted the importance of estrogen in the male, because sperm were found to be capable of estrogen synthesis [70–72]. Equally important was the report that E2 concentrations in rete testis fluid exceeded that of uterine tissues [58]. However, during that time period, it was assumed that loss of the hormone E2 or the receptor itself (ESR1) would result in similar pathologies. Therefore, the aromatase knockout (*Cyp19*KO) mouse phenotype [109, 167], which was not equivalent to that of *Esr1*KO males [100, 124, 125, 168], was perplexing. *Cyp19*KO males were fertile initially but showed progressive infertility with aging, as problems with spermatogenesis appeared, as well as altered sexual behavior [169–171]. Further study of these males revealed that dietary soy-containing phytoestrogens stimulated spermiogenesis, as *Cyp19*KO males fed soy-free diets had increased testicular pathology with aging [167]. However, to everyone's surprise, normal sperm were present in the cauda epididymis in the younger *Cyp19*KO males and the male reproductive tract was also normal [171, 172].

Efforts to unravel this *Cyp19*KO mystery are ongoing, but a definitive explanation has proven elusive. However, at least three areas of research have helped to provide plausible explanations for the lack of efferent ductule abnormalities following the loss of endogenous estrogens:

1. Subsequent studies found that ESR1 expression in *Cyp19*KO efferent ductules was retained and equaled that of wild-type males [142]. Therefore, the *Cyp19*KO phenotype could reflect a hormone-independent (ligand-independent) transcriptional activation of the unliganded ESR1, which was conserved in this tissue. If the receptor is present, even in the absence of estrogen, ligand-independent signaling could occur via growth factor signaling and phosphorylation of the N-terminal AF-1 (activation function-1) domain [173–175]. In support of this argument, a knock-in mouse model (ENERKI) was developed with a mutated ligand-binding domain in ESR1 (Table 1) [176, 177], and efferent ductules were found to be normal, although the testes showed degeneration with aging, similar to *Cyp19*KO males. However, a more recent mutant ESR1 model, the AF2ER (activation function-2 domain) mutant that also targeted the LBD (Table 1), was found to be infertile and exhibited the *Esr1*KO male phenotype [144, 178]. It should be noted that the AF2ER phenotype was corrected by treatment with tamoxifen, which only worked as an AF-1 (ligand-independent domain) agonist in this model. Therefore, the precise mechanisms involved in regulation of efferent duct gene transcription through both AF-1 and AF-2 are more complex than first anticipated and may involve unique cell and tissue specific interactions [179].
2. ESR1 expression is constitutive in efferent duct epithelium, with intense ESR1 immunostaining even after castration. ESR1 was downregulated only by high E2 doses [160]. Therefore, if ESR1 expression was retained in *Cyp19*KO efferent ductules, a testosterone metabolite, such as 5α-androstane- 3β-17β-diol (3β-diol), which does not bind AR but binds ESR1 and ESR2 (with higher affinity for ESR2) [180–182], could mediate ESR1 activity and maintain epithelial function. Indeed, 3β-diol was later found to be as effective as E2 in maintaining postcastration efferent duct function [183] and thus could be involved in the *Cyp19*KO response.
3. Finally, dual regulation of efferent duct fluid reabsorption by AR and ESR1 is another plausible interpretation. Although hormone response elements are not always essential for steroid receptor transcriptional activation, key genes involved in epithelial maintenance and fluid reabsorption in efferent ductules, such as *Slc9a3* (NHE3) and *Slc9a3R1* (NHERF1), contain both EREs and AREs in their promoters and thus may be coregulated by both steroid receptors [154–156]. In *Cyp19*KO mice, androgens were still present and AR and ESR1 expression remained intact [142]. Therefore, the importance of an estrogen/androgen balance that requires both steroid receptors may explain some complexities observed in the regulation of fluid reabsorption in efferent ducts [164, 184].

## Membrane ESR1 is essential in normal male reproductive tract development and function

In the early 1960s, the search for mechanisms of steroid hormone actions was just beginning, but in 1968 Bert O’Malley published the first evidence to show that estrogen and progesterone act in the nucleus at the genomic level to induce transcription of new proteins [185, 186]. This model for actions of E2 and other steroid hormones became the predominant focus of the vast majority of the investigators in this field interested in the mechanisms of steroid hormone effects.

Despite the strong focus of this field on genomic actions, another literature has developed over a period of decades documenting rapid effects of steroids that did not appear to be nuclear or at the transcriptional level, but rather in the cytoplasm or cell membrane. This literature goes back to the mid-20th century. The original description of rapid steroid effects was by Hans Selye, also known for his description of the General Adaptation Syndrome [187]. Selye's initial studies were conducted with glucocorticoids, but rapid actions of other steroids, notably estrogens, were also described. Rapid actions of E2 were identified by Claire Szego at the University of California-Los Angeles in the 1960s and then explored in a series of landmark papers that were critical in establishing the concept of membrane receptors for steroid hormones in general and for estrogen in particular [188–190].

A critical step forward in this field was the demonstration that membrane binding of E2 resulted from the same ESR1 molecule that mediated its genomic actions. Localization of ESR1 to the membrane following synthesis was dependent on palmitoylation of cysteine 451 in the E-domain of the mouse ESR1 molecule [191, 192]. In the absence of palmitoylated cysteine 451 in mouse ESR1, membrane localization of the receptor molecule was prevented, and the entire cellular ESR1 complement was nuclear. This major step in understanding trafficking of newly synthesized ESR1 to the cell membrane provided a powerful tool for ultimately producing mice lacking mESR1.

Development of the homologous recombination technique by Oliver Smithies allowed production of mice with a particular gene disabled, or knocked out. This technique had and continues to have major impacts in many fields of biology, and Smithies, along with Mario Capecchi and Martin Evans, received the Nobel Prize in Physiology or Medicine in 2007 for their discoveries that paved the way for the development of knockout mice. The first application of this technique in the steroid receptor field was the report in 1993 by Dennis Lubahn, Oliver Smithies, and Ken Korach of mice with *Esr1* knocked out. This original *Esr1*KO mouse [124] and a subsequent version from Pierre Chambon and Andrée Krust in France [193] have been used extensively for the last 25 years and are among the most powerful tools in reproductive biology.

Knockout of the steroid receptor or the enzyme involved in estrogen production (e.g., aromatase) [194] opened new vistas for investigation (Table 1). Despite extensive data gathered literally over decades indicating that mESR1 was important for normal E2 responses, mESR1’s role in males and females was unclear. Even with widespread utilization of knockout mice in steroid endocrinology, it was not initially possible to harness this powerful technique to study mESR1, because the mESR1 in a cell represented just a small component of the overall receptor in that cell and there was no method to knock out mESR1 while maintaining nESR1.

The seminal discovery that palmitoylation at one specific site in the mouse ESR1 molecule was required for membrane localization provided an opportunity to produce transgenic mice lacking mESR1. Two separate transgenic mouse lines with alanine substituted for cysteine at position 451 in ESR1 were described in 2014 [195, 196]. Replacement of the normally palmitoylated cysteine 451 with alanine (which cannot be palmitoylated) impaired membrane ESR1 localization despite continued presence of normally functional nESR1. Interestingly, despite similar approaches employed by both groups to make transgenics lacking mESR1, Adlanmerini et al. [196] reported that mESR1 was reduced only by approximately 50% in E2-target organs of their mouse, while Pedram et al. [195] showed that mESR1 was essentially absent in E2-target organs of their nuclear-only estrogen receptor (NOER) mouse. There were also other phenotypic differences, with Pedram et al. [195] reporting a uterine phenotype not seen by Adlanmerini et al. [196], which may have resulted from more complete elimination of mESR1 in the former.

The NOER mice provided a unique tool to determine whether mESR1 loss resulted in male reproductive changes. The infertility and reproductive effects in female NOER mice [195] suggested that male reproductive effects might also be found. The male NOER mouse phenotype [140] turned out to be strikingly similar to the *Esr1*KO male, although reproductive abnormalities were typically less severe in NOER compared to *Esr1*KO males. One hallmark of *Esr1*KO males is rete testis enlargement due to impaired fluid resorption by efferent ductules and consequent backpressure into the rete. Rete testis area of adult NOER males was increased 20-fold over normal control males, and was comparable to that in *Esr1*KO males. Degeneration of the seminiferous epithelium and enlargement of seminiferous tubules were also seen in NOER males, again paralleling *Esr1*KO males, although the NOER phenotype was less severe. The NOER males also had 85% decreases in sperm motility, 60% decreases in adult daily sperm production, and extensive sperm structural abnormalities. All of these occur in *Esr1*KO mice, although decreased sperm motility and sperm production are more severe in *Esr1*KO males. The NOER mice had decreased efferent ductule epithelial height and increased efferent ductule luminal diameters. Initial studies in 5-month-old adult NOER males revealed that, like *Esr1*KO males, they were infertile. However, since loss of mESR1 leads to progressive pathology, fertility of young NOER males was tested. Somewhat surprisingly, juvenile NOER males were often transiently fertile during a short window of time after puberty. Thus, NOER males show a progressive infertility, which is a less drastic phenotype than the totally infertile *Esr1*KO males.

Only approximately 5–10% of cellular ESR1 is mESR1. However, loss of mESR, whose importance was questioned for decades as the field focused on nESR1, leads to infertility and other male abnormalities. These findings, two decades after initial studies indicating that *Esr1*KO males were infertile due to abnormalities in the efferent ductules and other organs [100, 124, 125], further emphasized the critical role of estrogen in male reproduction. While initial studies with *Esr1*KO males and a man lacking ESR1 [197] in the 1990s indicated that the estrogen pathway was critical for development and/or function of some male organs in men and animals, the NOER results indicated that mESR1, in addition to nESR1, is essential for normal male development.

## Role of estrogen signaling in the developing and adult prostate gland

The prostate expresses ESR1 and ESR2 during development and adulthood, and early estrogen treatments produce deleterious effects on growth and development of the prostate and other accessory sex organs. Due to this, the prostate was examined in studies to characterize both *Esr1*KO and *Esr2*KO males. Some differences were identified in prostates of both *Esr1*KO and *Esr2*KO males [182, 198, 199], but prostatic phenotypes observed in these knockout mice were less severe than in organs such as efferent ductules of *Esr1*KO males [100, 124, 125]. This suggests that loss of ESR1 and ESR2 signaling may not be as critical for prostatic development as it is for other male reproductive tract organs, but work over the past few years has suggested that E2/ESR1 signaling could play an important role in major prostatic pathologies.

Early treatments of rodents with the potent synthetic estrogen DES and other natural and xenoestrogens produce permanent alterations in prostatic development and differentiation, and is referred to as developmental estrogenization [36, 200, 201]. As developmentally estrogenized animals age, their prostates show chronic inflammation, epithelial hyperplasia and adenomas, and neonatal estrogen treatment alters adult responsiveness to trophic hormones [202]. Developmental estrogenization effects are mediated through ESR1 [36, 37, 203], with both stromal and epithelial ESR1 necessary for a full DES response [37]. Prostatic changes induced by DES appear to involve epigenetic effects [204]. Although the mechanism of this effect is still being established, current data indicate that early estrogen exposure may reprogram prostatic stem and progenitor cells and alter their proliferation [153].

Estrogen has been shown to be involved in the most common human prostatic disease, benign prostatic hyperplasia [205]. The ability of early estrogen exposure to alter prostatic stem and progenitor cells and the involvement of estrogens in the etiology of benign prostatic hyperplasia suggest the possibility that developmental exposure to estrogens could induce changes that alter aging susceptibility to this hyperplastic growth and potentially other diseases such as prostatic cancer. Thus, future advances in understanding of estrogen signaling in prostate may provide insights into clinically important diseases, and this area is currently a focus of active investigation.

## Summary

A paradigm shift has occurred in reproductive biology regarding the role of estrogen in the male reproductive tract. We have gone from a belief that estrogen in the male was at best a remnant leftover from the indifferent sex stage of embryological development and at worst a hormone that induces developmental abnormalities to the current acceptance that estrogen and ESR1 are essential for male fertility and reproductive function. Despite significant advances in our understanding of estrogen's importance in the male reproductive tract, many questions remain unanswered, and future work will likely reveal further unexpected insights into the role that estrogens play in the male.
